# Edinburgh Royal Infirmary and Approved Societies

**Published:** 1922-04

**Authors:** Wm. S. Caw

**Affiliations:** Treasurer and Clerk, The Royal Infirmary, Edinburgh


*To the Editor of* THE HOSPITAL AND HEALTH REVIEW.

### Edinburgh Royal Infirmary and Approved Societies.

*To the Editor of* THE HOSPITAL AND HEALTH REVIEW.

SIR,—My attention has been called to the paragraph bearing the above heading in your issue of this month (March). Let me say (1) that no Approved Society has intimated a declinature to subscribe on the grounds suggested in that paragraph, and (2) that Approved Societies have subscribed, although not in any sense to the extent my Board felt justified in anticipating.

With regard to the question of certificates, these have always been granted free. The misleading statement as to the position of this hospital originated in an anonymous letter published in one of the Edinburgh evening newspapers on December 1 last. Our Superintendent sent a reply thereto on the following day, which was duly published, and I cannot do better than quote the following paragraph from it :—

"Any 'Insured Person' who may be a patient in this Institution has only to apply to the Sister of the Ward in which he is being treated, for a certificate (which is granted *free*), as often as his Approved Society requires it. This certificate is quite sufficient under Memorandum No. 211, paras. 38 and 39, issued by the Insurance Commissioners, to enable any Approved Society to pay sick benefit to their members. The fee of one guinea referred to only applies to cases where claims are made under the Workmen's Compensation Acts, or the Employers' Liability Acts, or at Common Law, and has nothing whatever to do with the National Health Insurance Act."

A few words of explanation as to the Royal Infirmary's relationship to the National Insurance Act may not be out of place at this time. As you are aware, the hospital has always been run on the "open-door" principle, and it has dealt with insured patients exactly as with those not benefiting under the Act. No claim has ever been made on its behalf for the sick benefit of the insured patients, without dependents, who have been treated. After the Act has been in force for some time, an appeal for support was issued to the Approved Societies, whose members had received the benefits of the Institution. This was met, all round, by the reply that nothing could be done until after the first actuarial investigation had been completed and the financial position of each Society ascertained. Early in 1921 the appeal was renewed to 196 Societies, accompanied by detailed schedules (a print of which I enclose) showing what had been done for insured persons during the five years to October 1, 1918. As you will observe, the cost of maintenance of these patients, calculated on the average outlay per bed in each year, gave a total of over £148,000 in the five years. Response to that appeal so far has been limited to 18 donations, amounting in all to £1,784, which includes sums from individual Societies of £700, £250, and two of £200 each. I refrain from commenting upon these facts, preferring to leave that to you.

I am, yours faithfully,
WM. S. CAW,
Treasurer and Clerk,
The Royal Infirmary, Edinburgh.

[We welcome Mr. Caw's explanation, which, as we expected, puts the matter in its proper light. It is also useful to have the statistical value of the services performed by the hospital for the Approved Societies. Inadequate as the reply to its appeal to them has been, the principle of their obligation has been accepted, and we cannot but think that it will be more fitly acknowledged before long.—ED.]

